# Feasibility of patient‐specific quality assurance (PSQA) for real‐time robotic stereotactic body radiotherapy (SBRT) based on tumor motion traces

**DOI:** 10.1002/acm2.14352

**Published:** 2024-05-02

**Authors:** Qianyi Xu, Jiajin Fan, Yevgeniy Vinogradskiy, Ashish K. Chawla, Gregory Kubicek, Haihua Yang, Kiet Huynh, Tamara LaCouture, Jimm Grimm, Wei Nie

**Affiliations:** ^1^ Department of Advanced Radiation Oncology and Proton Therapy Inova Schar Cancer Institute Fairfax Virginia USA; ^2^ Department of Radiation Oncology Thomas Jefferson University Philadelphia Pennsylvania USA; ^3^ Department of Radiation Oncology University of Miami Miami Florida USA; ^4^ Department of Radiation Oncology Taizhou Hospital Taizhou Zhejiang China; ^5^ Department of Radiation Oncology Wellstar Health System Marietta Georgia USA

**Keywords:** CyberKnife, diode array, motion platform, PSQA, synchrony

## Abstract

**Purpose:**

To design a patient specific quality assurance (PSQA) process for the CyberKnife Synchrony system and quantify its dosimetric accuracy using a motion platform driven by patient tumor traces with rotation.

**Methods:**

The CyberKnife Synchrony system was evaluated using a motion platform (MODUSQA) and a SRS MapCHECK phantom. The platform was programed to move in the superior‐inferior (SI) direction based on tumor traces. The detector array housed by the StereoPhan was placed on the platform. Extra rotational angles in pitch (head down, 4.0° ± 0.15° or 1.2° ± 0.1°) were added to the moving phantom to examine robot capability of angle correction during delivery. A total of 15 Synchrony patients were performed SBRT PSQA on the moving phantom. All the results were benchmarked by the PSQA results based on static phantom.

**Results:**

For smaller pitch angles, the mean gamma passing rates were 99.75% ± 0.87%, 98.63% ± 2.05%, and 93.11% ± 5.52%, for 3%/1 mm, 2%/1 mm, and 1%/1 mm, respectively. Large discrepancy in the passing rates was observed for different pitch angles due to limited angle correction by the robot. For larger pitch angles, the corresponding mean passing rates were dropped to 93.00% ± 10.91%, 88.05% ± 14.93%, and 80.38% ± 17.40%. When comparing with the static phantom, no significant statistic difference was observed for smaller pitch angles (*p* = 0.1 for 3%/1 mm), whereas a larger statistic difference was observed for larger pitch angles (*p* < 0.02 for all criteria). All the gamma passing rates were improved, if applying shift and rotation correction.

**Conclusions:**

The significance of this work is that it is the first study to benchmark PSQA for the CyberKnife Synchrony system using realistically moving phantoms with rotation. With reasonable delivery time, we found it may be feasible to perform PSQA for Synchrony patients with a realistic breathing pattern.

## 1 INTRODUCTION

Stereotactic body radiotherapy (SBRT) has been widely used for lung and liver cancer treatment. Conformal and high gradient dose was delivered to lesions to achieve effective local tumor control and minimize side effects. The M6 CyberKnife (CK) system (Accuray Inc., Sunnyvale, CA), composed of a robot mounted x‐band linear accelerator (LINAC), 6D robotic couch and stereo‐imaging system, has been developed for SBRT utilizing a unique real‐time tracking system to deliver dose to moving tumors. Specifically, the M6 CK system with synchrony respiratory tracking system monitors the motion of implanted fiducials near a target through the kV imaging system and builds motion model by correlating the motion of optical markers (LEDs) attached to patient body with dynamic target/fiducials motion. With the correlation model, the Synchrony system predicts the moving target position to account for LINAC latency and radiation is delivered to the tumor in real‐time following tumor motion.^1^, ^2^ The correlation model is periodically updated by pairs of X‐ray image taken at a user‐defined interval, by which the predicted position from correlation model is verified. When the monitored LED markers deviate out of tolerance position, the beam is interrupted until a new model is established.

The CK SBRT PSQA has been performed with radiochromic film and ion chamber in solid water. Film provides 2D relative dose distribution at high resolution with absolute dose verified by an ion chamber.^3^, ^4^ The film method requires a skill set of film processing with delayed readout to achieve acceptable results.^5^ In recent years, several QA devices based on ion chamber/detector array have been developed to perform PSQA for SRS/SBRT plans.^6^, ^7^, ^8^, ^9^, ^10^, ^11^, ^12^, ^13^ These devices include 2D ion chamber arrays, such as PTW Octavius 4D (O4D) Modular Phantom (PTW, Freiburg, Germany) and MatriXX (IBA, Schwarzenbruck, Germany), and high resolution 2D detector arrays, such as IBA my QA SRS CMOS matrix (IBA Dosimetry GmbH, Schwarzenbruck, Germany) and SRS MapCheck 2D diode array (Sun Nuclear, Melbourne, FL, USA). Beside these, Delta4 (ScandiDos AB, Uppsala, Sweden) is an orthogonal planar semiconductor array embedded in a cylindrical acrylic phantom capable of interpolating measured dose points in 3D.^14^ All above devices have been validated for a variety of beam energies, dose rates, and angular responses to perform IMRT/SBRT patient QA on static phantoms.

While the technology for PSQA using static phantoms has been advanced, there has a lag in developing moving phantoms for CK Synchrony PSQA. Some pioneer studies have explored using respiratory phantoms when delivering Synchrony plans. Marants et al. delivered experimental Synchrony plans on a moving phantom and analyzed gamma passing rates based on film measurements.^15^ The number of cases was very limited and only one lung plan was delivered based on a patient tumor trace. Kawabata et al. performed CK Synchrony patient QA with SRS MapCheck on a motion platform driven by 1D sinusoidal signal.^16^ Both studies had some limitations. The film used in the study by Marants et al. needed extra processing with delayed readouts. In the study by Kawabata et al., the phantom was driven by a simple 1D sinusoidal signal, which could not mimic real patient breathing pattern. Thus, we proposed to combine SRS MapCheck with Quasar programmable motion platform (Modus Medical Devices, Ontario, Canada) for CK Synchrony PSQA. The motion platform was driven by patient tumor traces, on which the SRS MapCheck provided real‐time dose readout from its diode array. Furthermore, the Synchrony system was capable of providing intrafractional rotational motion correction after taking periodic pairs of X‐ray image. The rotational correction from the robot included 1.5° in roll and pitch and 3° in yaw. In our experiment, extra rotational angles in pitch were added to the moving phantom to examine robot capability of angle correction during delivery. Overall, the aim of the study was to (1) design and illustrate a PSQA process for CK Synchrony treatments and deliver a total of 15 lung and liver patients and (2) to evaluate dosimetric accuracy of the CK Synchrony system due to respiratory motion and rotation.

## 2 MATERIALS AND METHODS

### 2.1 SRS MapCheck and StereoPHAN phantom

**FIGURE 1:**
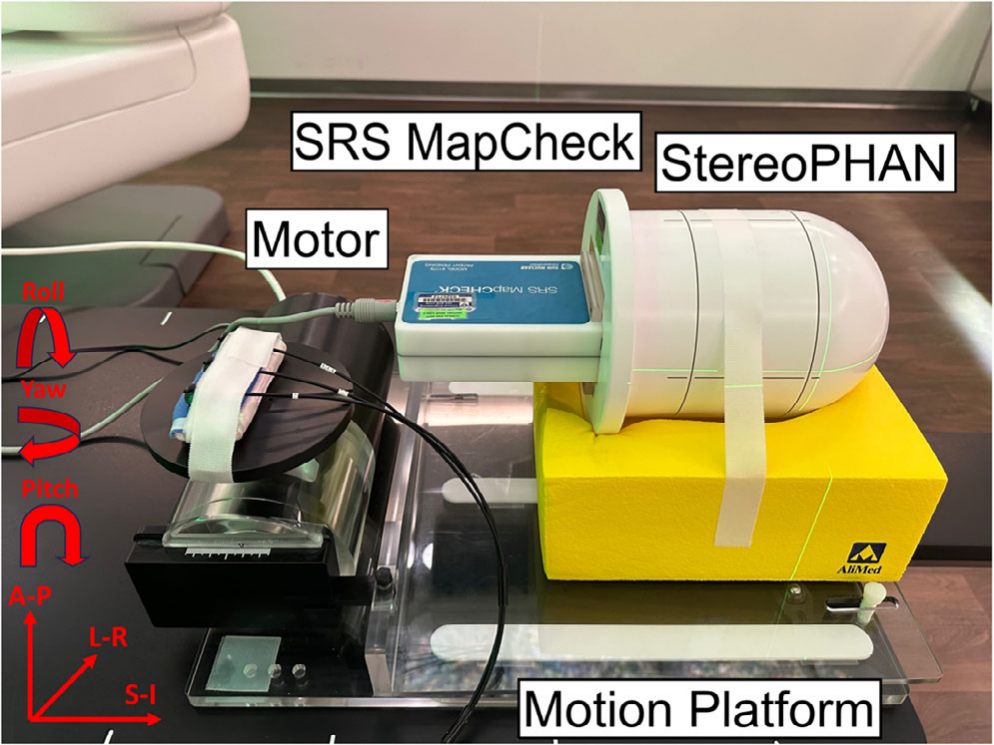
The SRS MapCheck and StereoPHAN on QUASAR motion platform. The phantom was placed on motion platform and fixed on a head rest.

The SRS MapCheck, with 1013 n‐type diode detectors, a cross‐section area of 0.48 mm × 0.48 mm for each unit and 2.47 mm apart between neighboring diodes, was suitable for LINAC SBRT/SRS PSQA for beam energies from 6 and 10 MV and field sizes from 5 mm × 5 mm to 77 mm × 77 mm.^13^ The diode array was sandwiched by two 22 mm polymethyl methacrylate (PMMA) slabs. There were four fiducials embedded around diode array for setup alignment. The SRS MapCheck, housed by the StereoPHAN (Figure 1), were scanned by GE Revolution CT following our CK protocol (120 kV, 500 mm FOV, 1.25 mm slice thickness and Smart mAs). The mass density of the whole unit was assigned to 1.2 g/cm^3^ per vendor manual. The array calibration supplied by the vendor was used in the study since it's not sensitive to beam energies. A single anterior‐posterior (AP) beam was delivered to the central area of the detector array and the dose calibration was acquired after the corresponding dose was derived from TPS. Other correction factors, including angular correction factors, dose rate correction factors, field size correction factors and temperature correction factors, were supplied by the vendor.

### 2.2 Quasar motion platform

**FIGURE 2:**
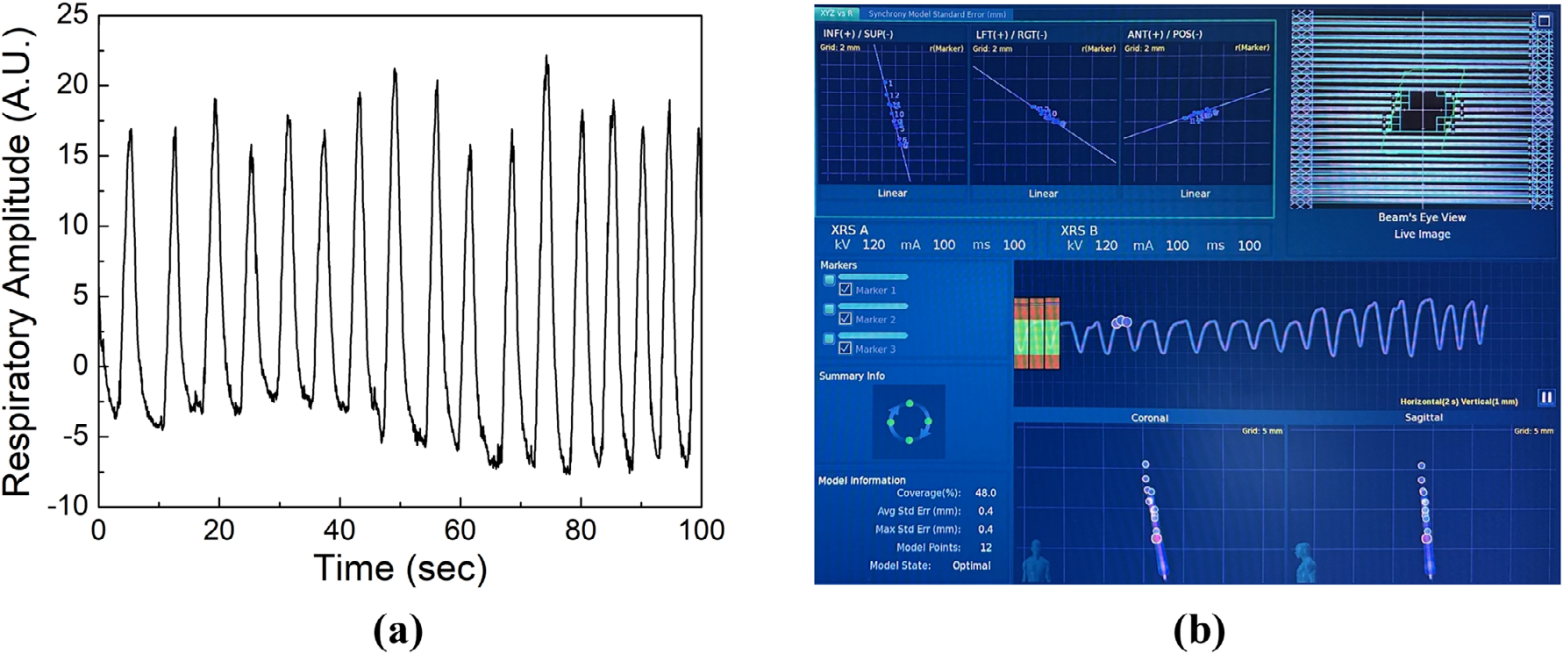
An example patient tumor motion trace in SI imported to Quasar motion platform (a) and signal tracked by LED markers during CK synchrony PSQA (b). The tracked both signals were not matched by the exact timestamp. The signal from LED markers was shown as the waveform (the middle row of (b)), representing maker breathing pattern and overlaying with Synchrony model points. In (b), the Synchrony motion model between the external LED markers and internal fiducials was displayed as correlation plots in SI, LR and AP (the top row of (b)). The “comet” graphs (the bottom row of (b)) showed qualitive pictures of how the model points fitted into the current Synchrony model.

The Quasar motion platform was capable of being programed and moving in the SI direction following a patient respiratory signal (maximal moving range: −15 mm to 15 mm and frequency range: 4–30 breaths per minute (bpm)). The platform has two major components: the control unit with a motor and the moving platform (Figure 1). In our study, the tumor motion traces, which replaced the respiratory signals, were retrieved from the log files after CK synchrony treatment. We adopted the tumor motion traces in the SI direction as the largest tumor motion occurred in this direction for lung and liver patients. The trace was smoothed with 1 Hz low pass filter and renormalized to the maximal phantom moving range (−15 mm to 15 mm). The processed signal was fed into the control unit to drive the motion platform. An example signal was shown in Figure 2(a) and its corresponding LED breathing signal was shown in Figure 2(b). Additionally, two swivel wheels were installed underneath the distal end of the platform to introduce an extra shift in AP. When setting up the phantom, we intentionally added an extra rotational couch angle in pitch (1.2° or 4.0°) and deliver the same plan separately. In summary, the phantom moved following the processed tumor motion trace in the SI direction, with extra shift in AP and a fixed rotation angle in pitch.

### 2.3 PSQA preparation and delivery

The clinical SBRT plans were optimized with the Volo inverse planning technique (Precision version 2.0.1.1) using MLC for delivery, following the dosimetric guidelines.^17^ Fractionation schemes from 40 to 54 Gy in 3 to 5 fractions were prescribed to the PTV at about 80% isodose line. The PSQA plans were created by overlaying the clinical plans with the SRS MapCheck/StereoPhan phantom plan. The PTV was aligned with the central portion of the detector plane and the alignment was fine‐tuned, if needed, to ensure four fiducials were visible in the X‐ray images. The QA plans were recalculated with high‐resolution dose grid using finite size pencil beam (FSPB) algorithm. The QA phantom on the Quasar motion platform was set up on the 6D couch (Figure 1). The static StereoPHAN was aligned with the room laser and further fine‐tuned by fiducial alignment after pairs of X‐ray images were taken. Afterwards, a pitch angle was manually introduced to the phantom by rotating the robotic couch using the teach pendent. For each PSQA, two different pitch angles (1.2° and 4.0°) were experimented separately to evaluate the impact of rotation angles on the PSQA results. Once a pitch angle was added, the motion platform started to move and PSQA delivery started. During delivery, the rotational correction function was turned on for the smaller pitch angle (1.2°) and rotation bound check only was activated for the larger pitch angle (4.0°) due to limited capability of angle correction from the robot. The PSQA dose was imported into SNC Patient software (version 8.5.1) and compared to the measured dose for gamma analysis. QA results were evaluated with criteria of 3%/1 mm, 2%/1 mm, and 1%/1 mm using 10% threshold, with and without applying additional shifts and rotation correction in the SNC software.

## 3 RESULTS

**FIGURE 3:**
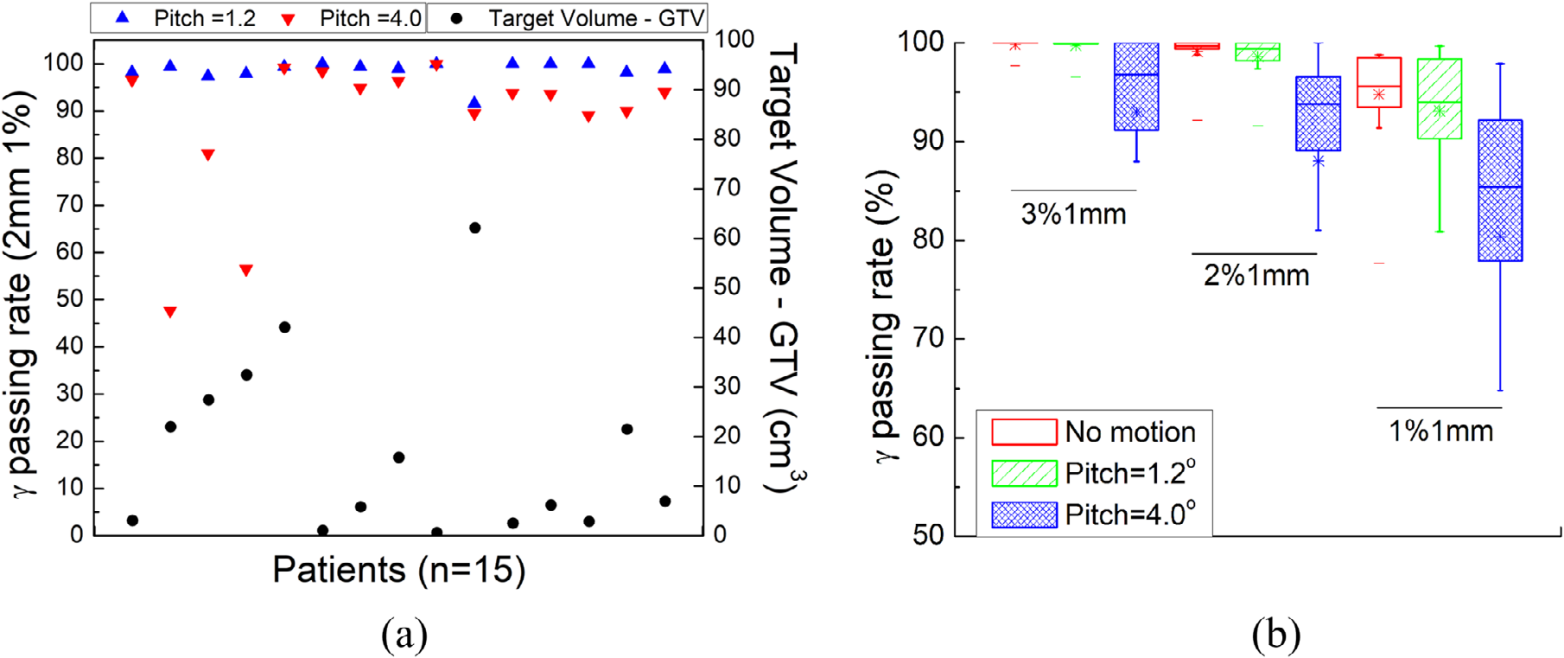
PSQA results with 2%/1 mm criterion from the moving phantom (a) and comparison of PSQA results between the static and moving phantom (b). The gamma passing rate was indicated as upward triangles for the pitch angle of 1.2° and downward triangles for the pitch angle of 4.0° (a). The target volume (GTV) was indicated by circles in (a). The mean gamma passing rates (error bar for standard deviation) from the static phantom and moving phantom for all three criteria were shown in (b).

A cohort of 15 Synchrony SBRT plans (14 lung and one liver) were selected to perform PSQA. The volume of the target (GTV) ranges in 1.02–62.12 cm^3^ (Figure 3a). The PSQA plan delivery time was 27.2 ± 4.4 min (mean ± standard deviation). The irradiation time for any specific plan depended on delivered dose, target size, shape, and irregularity of patient breathing pattern. The InCise 2 MLC had limited size of aperture (11.5 cm × 10.0 cm at 80 cm SAD) and targets with a larger size and higher dose needed more throughput from the machine. The shape of the target determined the modulation and segment size in the plan, which contributed significantly to the total treatment time. The patient breathing pattern was also an important factor on interrupt of treatment to rebuild the Synchrony model. Also, CK PSQA was routinely performed in our clinic for Synchrony patients using the static SRS MapCheck/StereoPHAN phantom. The QA results from the same cohort of patients were used as benchmark and compared with those from the moving phantom.

**FIGURE 4:**
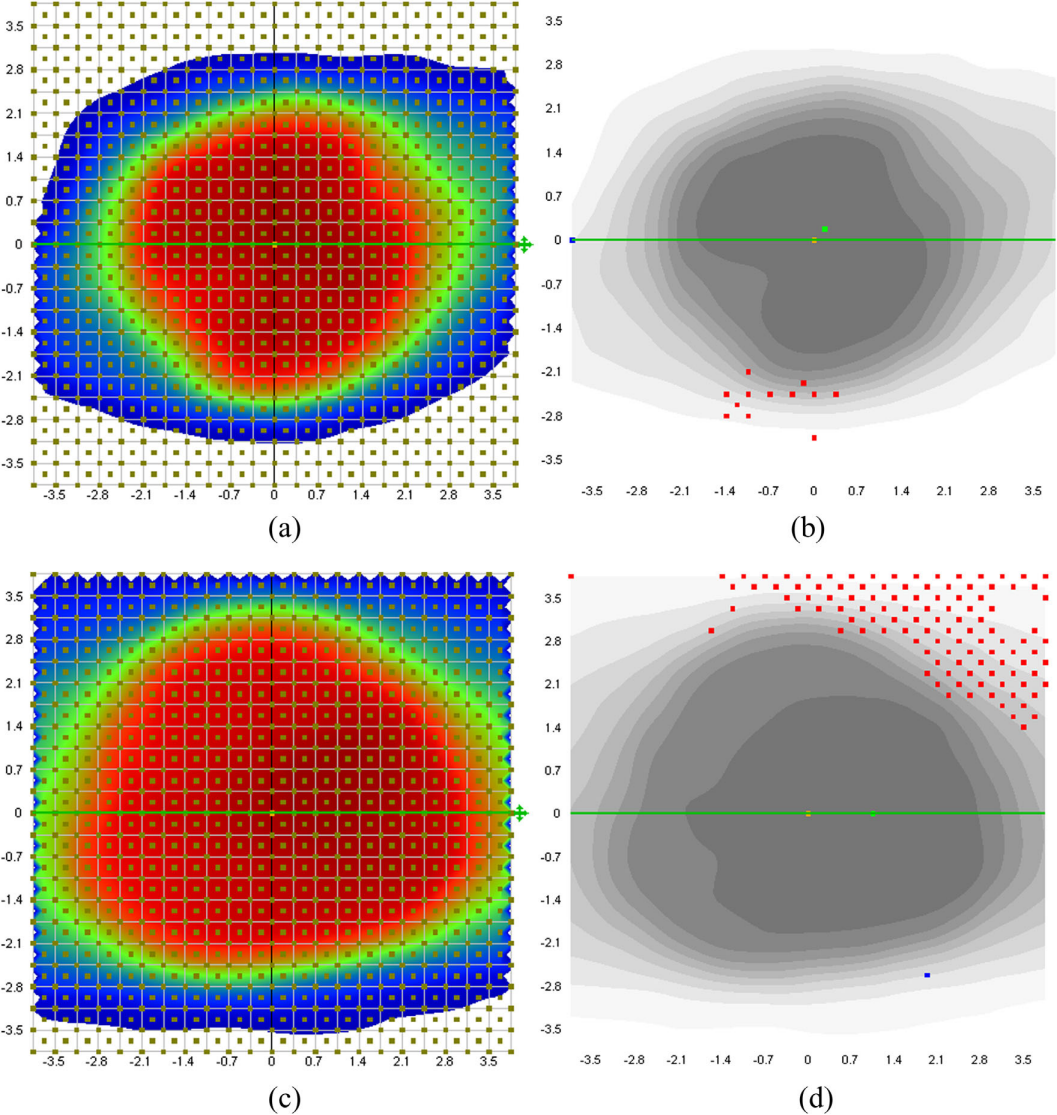
PSQA results for two patients (pitch angle of 4° and 3%/1 mm criterion) with high (top row) and low (bottom row) passing rates. The dots indicate the corresponding failed QA points.

The specific SBRT/SRS gamma passing criteria were not provided by AAPM TG‐218,^18^ although 3%/2% (dose difference) and 1 mm (distance to agreement) have been proposed with 95% passing rate and 90% action limit with 10% threshold.^19^ In this study, the criteria of 1%, 2%, and 3% and 1 mm with 10% threshold were adopted for PSQA evaluation. We first reported gamma passing rates without applying corrections from the SNC software. Figure 3(b) presented the average gamma passing rate for PSQA results from the static and moving phantom with either a small or large pitch angle (1.2° or 4.0°). For moving phantom with a small pitch angle, the 3%/1 mm gamma passing rate was 99.75% ± 0.87%, which was not significantly different from that from the static phantom (99.85% ± 0.59%, *p*‐value = 0.1). When the criteria were raised to 2%/1 mm and 1%/1 mm, there were statistically significant difference between the results from static and moving phantom (99.15% ± 1.89% vs. 98.63% ± 2.05% (*p*‐value = 0.02) and 94.80% ± 5.12% vs. 93.11% ± 5.52% (*p*‐value = 0.04)). However, the passing rates from majority cases with moving phantom were still > 90% with tighter criteria since robot corrected the small pitch angle during delivery. For moving phantom with a large pitch angle of 4°, the gamma passing rates were reduced to 93.00% ± 10.91%, 88.05% ± 14.93%, and 80.38% ± 17.40% for 3%/1 mm, 2%/1 mm, and 1%/1 mm, respectively. These were significantly lower than the passing rates from static and moving phantom with a smaller pitch angle (*p*‐value < 0.02 for all criteria). The passing rates (2%/1 mm criterion) for all 15 cases were plotted in Figure 3(a), with significant difference observed between different pitch angles. Figure 4 showed QA results from two patients (pitch angle of 4°, one with high passing rate of 98.2% (Figure 4, top row) and one with low passing rate of 88.0% (Figure 4, bottom row), 2%/1 mm criterion). The corresponding acquired dose distributions were shown in Figure 4(a, c) and failed points (gamma passing rate > 1) were shown in Figure 4(b, d). The failed points occurred mostly in the superior and inferior ends for both patients. This would be understandable that since the robot couldn't correct for the large pitch angle, the offset was the largest near the superior and inferior ends. The case with lower passing rate was likely due to the larger tumor size in the SI direction with more offset in the superior end.

**FIGURE 5:**
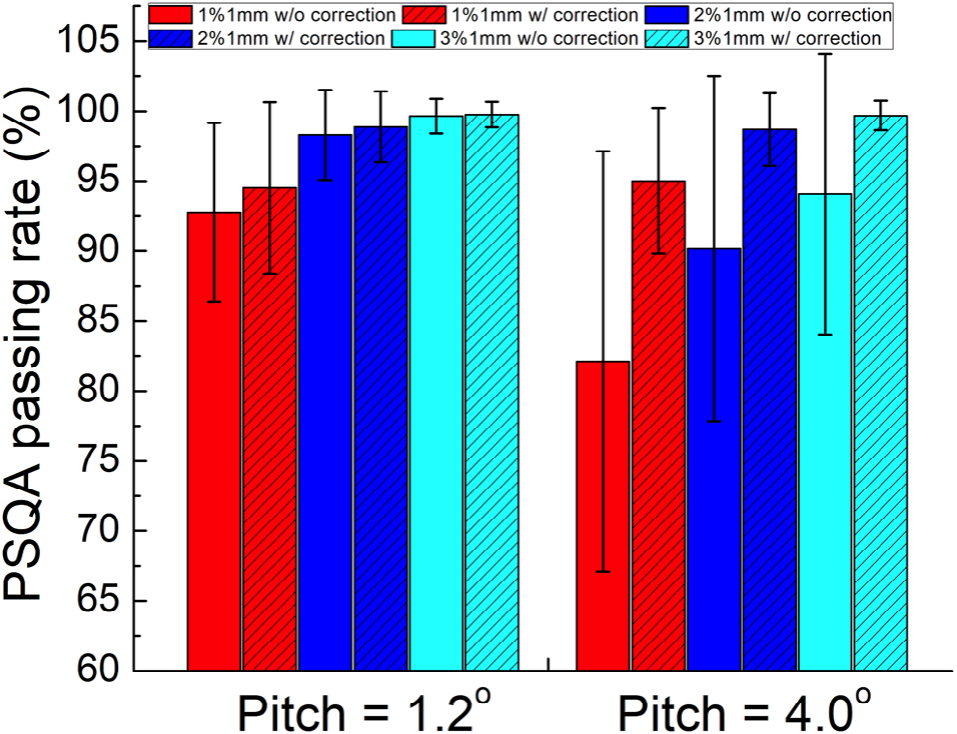
PSQA passing rate with and without correction in x, y, z and roll, yaw, and pitch with different criteria.

**FIGURE 6:**
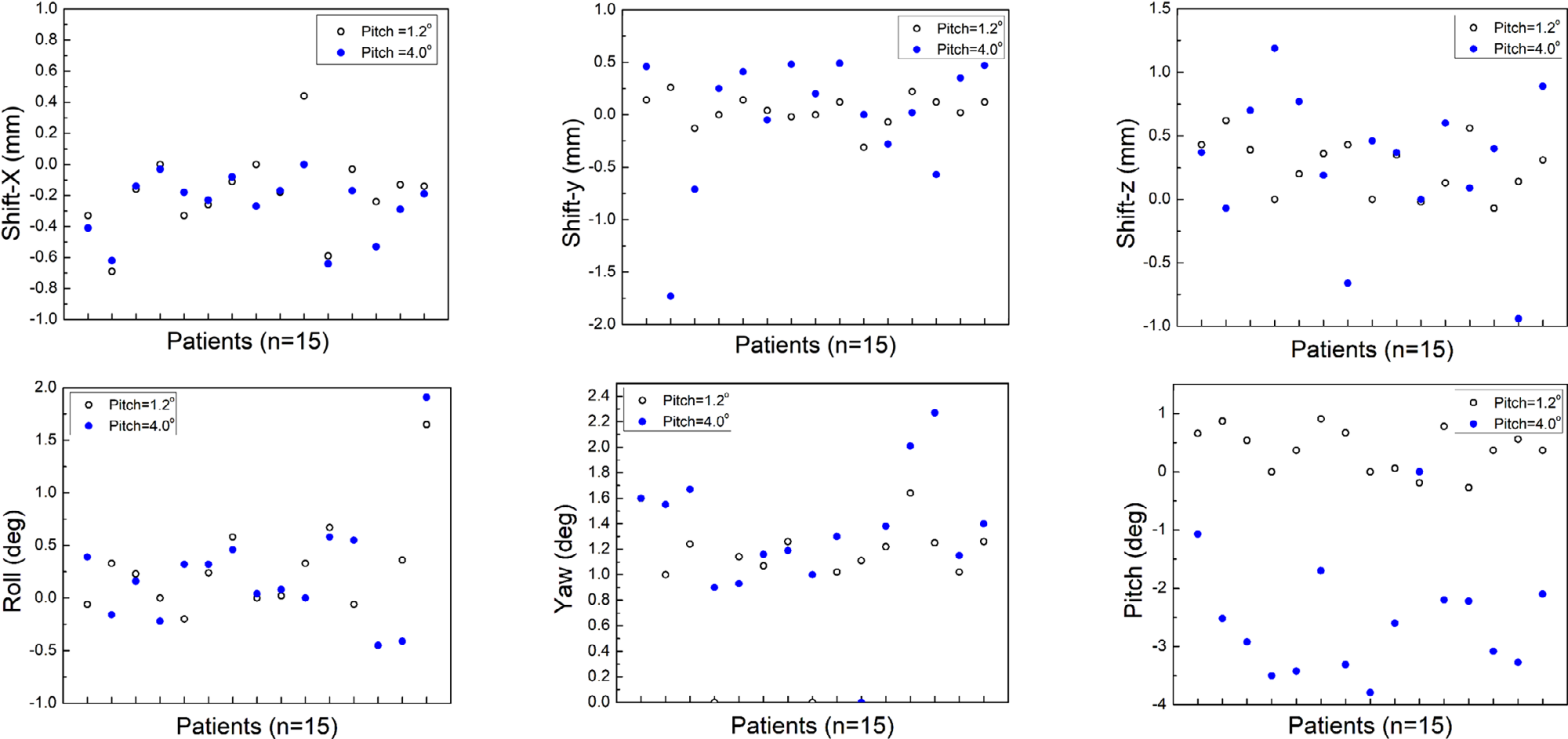
The reported shifts and rotation angles from SNC Patient software to maximize the gamma passing rates. The consistent translational corrections in x, y, z direction were shown in the top row. The rotational corrections in roll, yaw and pitch were shown in the bottom row. Open and solid circles correspond to pitch angles of 1.2° and 4.0°, respectively.

After applying the correction from the SNC software, the gamma passing rates were overall improved and the degrees of improvements were highly dependent on the pitch angle applied to the phantom (Figure 5). For pitch angle of 4°, the mean gamma passing rates were significantly improved to 99.69% ± 1.04%, 98.69% ± 2.60%, and 95.02% ± 5.21% with 3%/1 mm, 2%/1 mm, and 1%/1 mm criteria (v.s. 94.08% ± 10.04%, 88.0% ± 14.9%, and 82.11% ± 15.02% before correction). The reported mean shift and rotation corrections were −0.26 ± 0.19 mm, −0.01 ± 0.59 mm, 0.29 ± 0.54 mm in x (LR), y (SI), z (AP) direction, and 0.24 ± 0.55°, 1.30 ± 0.51°, −2.51 ± 0.98° for roll, yaw and pitch (Figure 6). The largest correction occurred in pitch (Figure 6f), which was expected since the large pitch angle was not corrected by the robot during delivery. For pitch angle = 1.2°, the mean gamma passing rates after correction were 99.76% ± 0.90%, 98.88% ± 2.53%, and 94.51% ± 6.12% with 3%/1 mm, 2%/1 mm, and 1%/1 mm criteria (v.s. 99.64% ± 1.24%, 98.30% ± 3.21%, and 92.77% ± 6.38% before correction). Much smaller improvement was observed since the robot corrected the angle in pitch during delivery. The corresponding mean shift and rotation corrections were small too (0.18 ± 0.25 mm, 0.04 ± 0.14 mm, 0.26 ± 0.21 mm in x, y, z direction, and 0.24 ± 0.47°, 1.06 ± 0.4°, 0.38 ± 0.37° for roll, yaw, and pitch). The correction was also applied to the results from static phantom (mean passing rates after correction: 99.85% ± 0.57%, 99.15% ± 1.89%, and 94.80% ± 5.12%) and no significant difference was observed comparing with those from small pitch angle.

## 4 DISCUSSION

In this study, we performed PSQA for 15 CK Synchrony patients using SRS MapCheck phantom on a moving platform driven by patient tumor traces with additional pitch angles. Similar studies were rare due the challenging of including a moving phantom for CK PSQA. In the study by Marants et al., an experimental plan was delivered to the film inserted in the lung phantom driven by a patient tumor trace. The experimental plan consisted of a 15 mm cone (43 beams) and a 30 mm cone (28 beam) with a maximal dose of 276.5 cGy. The gamma passing rates were 81% (3%/1 mm) and 94% (3%/2 mm), which were lower than ours. The potential reasons could be the uncertainties introduced during film processing and 0.3 s of hysteresis effect added in the tumor trace. The hysteresis effect could be partially corrected by the Synchrony system if a non‐linear correlation model was chosen. Kawabata et al. performed a similar study using SRS MapCheck on a moving phantom with 1D sinusoidal motion (maximal amplitude of 4 cm in SI). Both SRS MapCheck and film measurements were made by delivering an experiment plan using MLC with a prescription dose of 13.5 Gy. Correction was made in the SNC software to maximize the gamma passing rate. They reported > 95% passing rates with 3%/1 mm criteria for both film and SRS MapCheck measurements, which were in line with our findings.

Here we summarized potential sources of errors associated with SBRT/SRS PSQA using SRS MapCheck. In the study performed by Rose et al., 84 SBRT/SRS QA plans were delivered on LINAC using SRS MapCheck at nine institutions. The major sources of errors were coming from coarse dose grid, output factors, poor CBCT image quality and setup. In our experiment, the output was verified as within 1% difference from monthly calibration. TG 101 recommend the dose grid used for SBRT plans should be less than or equal to 2 mm.^17^ Medical Physics Practice Guideline 9a also recommend dose grid space of 1.25 mm for SRS TPS calculation.^20^ The dose grid employed in our Precision TPS calculation was 0.63 mm × 0.63 mm × 1.25 mm for the QA plan. In Rose et al., the guideline from individual institution was adopted for setup—either laser or CBCT alignment. In one institution, they noticed CBCT imaging isocenter was offset from the mechanical isocenter. In our study, fiducial alignment was used for image guidance since four fiducials were embedded in the SRS MapCheck. The accuracy of fiducial alignment was verified (<0.95 mm) during daily Automated Quality Assurance (AQA) test and monthly fiducial and Synchrony end to end (E2E) QAs. Furthermore, the Synchrony system was capable of providing intrafractional motion correction, which included rotational correction (1.5° in roll and pitch and 3° in yaw) and shifts in SI, AP and LAT. The system also adaptively updated the correlation model, enabling the robot to accurately lock the target when drifting or change of breathing pattern occurred. We had to pointed out that the rotational correction from the Synchrony system was limited and its impact on PSQA results was also evaluated. Two mandate angles (1.2° and 4.0° in pitch) were added separately to the SRS MapCheck when delivering PSQA for all the patients. When pitch angle was 1.2°, the passing rate (3%/1 mm) was not significantly different from that from static phantom, whereas it was much worse when the pitch angle increased to 4°. The tumor rotational motion could be a significant factor affecting PSQA results due to limited rotational correction from the robot. The other factor could contribute to PSQA was the speed of tumor movement or breathing cycle. The average of breathing cycles was correlated to the PSQA gamma passing rate when pitch angle of 4.0° was added. For three PSQAs with gamma passing rates < 90% (3%/1 mm), the mean breathing cycle was less than 4 s (3.76, 3.68, and 3.66 s). For the rest of 12 PSQAs, the breathing cycle was 5.30 ± 1.91 s with gamma passing rate of 97.6 ± 2.7% using the same criteria. Previous study has also observed a higher passing rate at a respiratory cycle of 6.0 s than that of 4.0 s.^21^ This would be a good clinical suggestion that instructing patients for a slower breathing pattern could warrant more accurate dose during CK Synchrony delivery.

We had to admitted there were a few limits in the design of our study. First, the phantom was driven by patient tumor traces but didn't move exactly as tumors moved. The initial platform was designed to move only in the SI direction with a range of ±1.5 cm. though some tumor near the diaphragm could move beyond this ±1.5 cm range.^22^ We also observed tumor drifting beyond the range during treatment. For all these scenarios, the motion of the tumors had to be normalized within ±1.5 cm in our study. For some tumors in the upper lobe (<±1.5 cm movement in SI), we also normalized it to maximal range of ±1.5 cm so to examine the robot's capability to track larger tumor movements. By adding two swivel wheels underneath the distal end of the platform, a small movement in the AP direction was also introduced. Second, we retrieved the tumor breathing trace in CK logfiles and designed the phantom and platform for CK Synchrony PSQA in the retrospective study. For routine clinic, it would be hard to obtain patient tumor traces before treatment and the earliest would be after the first treatment. In some clinic, the simulation was performed in the CK room to form vac loc and a few X‐ray pairs were taken to examine fiducial visibility and migration before CT scans. In this case, a Synchrony model could be built to simulate patient treatment and the tumor trace could be retrieved before treatment or using LED motion to drive the moving phantom. Lastly, the fiducial geometry during patient treatment was much more complicated than those in the phantom. We have seen rigid‐body errors constantly reported during Synchrony treatment, indicating the distances between fiducials were not fixed due to elastic lung tissues.^22^ The fixed fiducials in the SRS MapCheck assumed an ideal fiducial geometry, which would not be often seen during treatment.

In this study, two mandatory fixed rotation angles were applied separately to the phantom during delivery. The aim of this design was to: (1) to examine the capability of robot angle correction and (2) to evaluate the impact on gamma passing rates when rotation angles were beyond ranges of robot correction. AAPM TG – 135 suggested that a known amount of offset should be applied to the phantom to examine the capability of robot shift and angle correction during E2E test. Subedi et al. added intentional shifts (up to 1 cm) and rotational angles (up to 1°) during CK daily AQA test.^23^ Their results showed submillimeter delivery accuracy was achieved in daily AQA test for more than 99% of the time over a span of 17 months. Our Synchrony PSQA delivery was much more complicated than AQA tests since the real patient plans were delivered on a moving phantom driven by tumor traces. Superior gamma passing rates were achieved in our experiment based on different criteria. Future efforts should be focusing on investigating delivery accuracy based different combinations of rotation angles. During the PSQA delivery, we also applied the rotation angle beyond the range of robot correction (for our RoboCouch system, CyberKnife was able to correct rotation angles <1.5° in roll and pitch and <3° in yaw). As expected, inferior gamma passing rates were observed, comparing with the static and moving phantom with correctible rotation angles. We also had to admit that the angles induced in the study couldn't represent real tumor rotation since varying tumor rotation angles would be expected during patient delivery. We expect future endeavor on developing phantoms with real tumor rotation would facilitate investigation on its impact on delivery accuracy.

## 5 CONCLUSIONS

The significance of this work is that it is the first study to benchmark PSQA for the CyberKnife Synchrony system based on a cohort of 15 SBRT patients using a realistically moving phantom. With reasonable delivery time, we found it may be feasible to perform PSQA with a realistic breathing pattern for Synchrony patients with superior gamma passing rates. For correctible rotation angles, superior gamma passing rates were observed due to robot intrafractional rotation correction. Special attention needed to be paid to angles beyond robot correction range since inferior passing rates were observed, though still met TG 218 recommendations.

## AUTHOR CONTRIBUTIONS

Qianyi Xu and Wei Nie contributed to the conception, design and analysis of the study. The first draft of the manuscript was written by Qianyi Xu and the rest of authors commented and edited the manuscript. We confirm that all coauthors contributed to the study.

## CONFLICT OF INTEREST STATEMENT

The authors declare no conflict of interest.
